# Supplementary figures and images for: Long-Term Evaluation of Dip-Coated PCL-Blend-PEG Coatings in Simulated Conditions
Source: Polymers (Basel). 2020 Mar 24;12(3):717. doi: 10.3390/polym12030717 (PMC7183267; doi:10.3390/polym12030717)

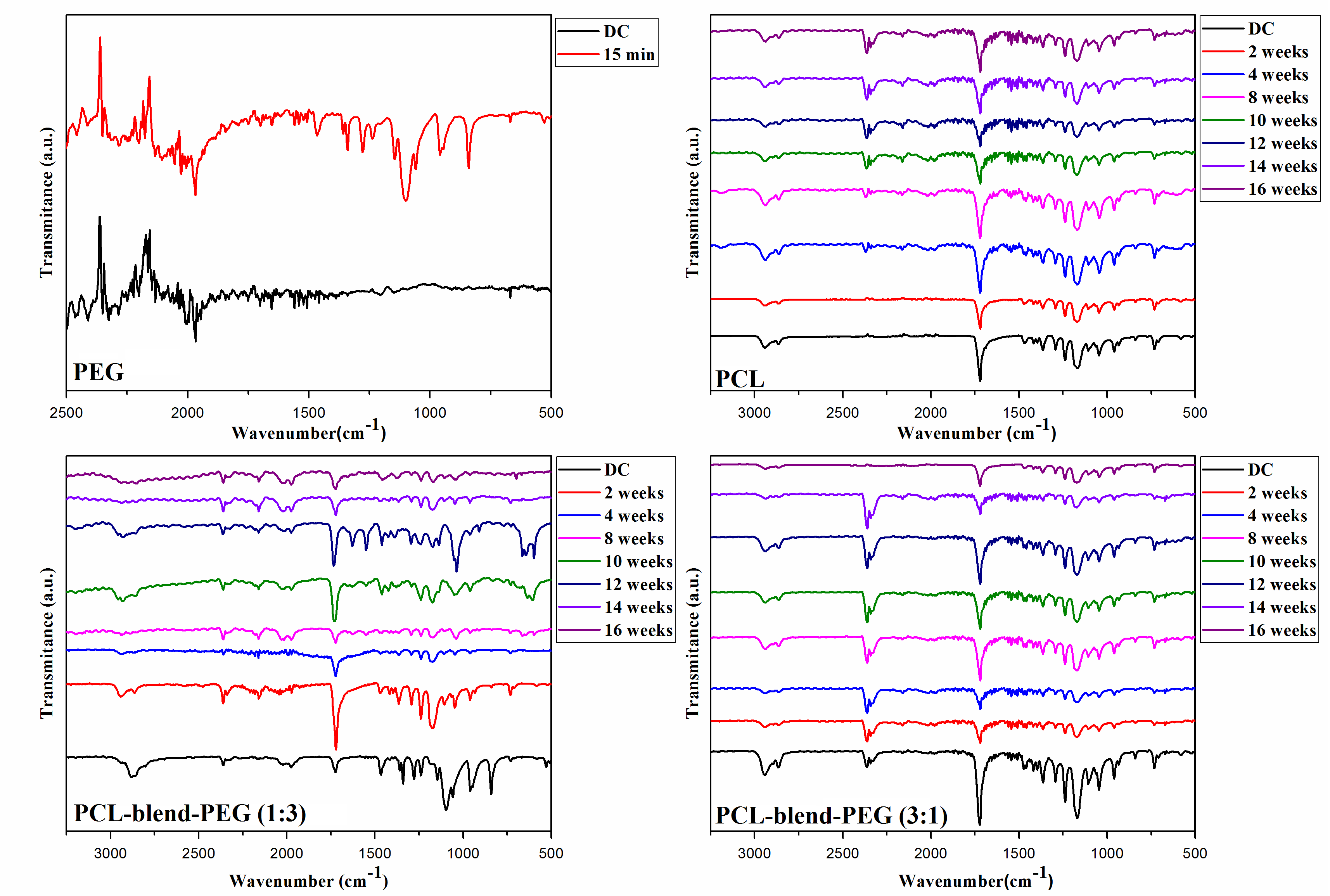

Supplement: Supplementary file 1 [file polymers-12-00717-s001.zip › Supplementary figures/ATR suplementary.tif]

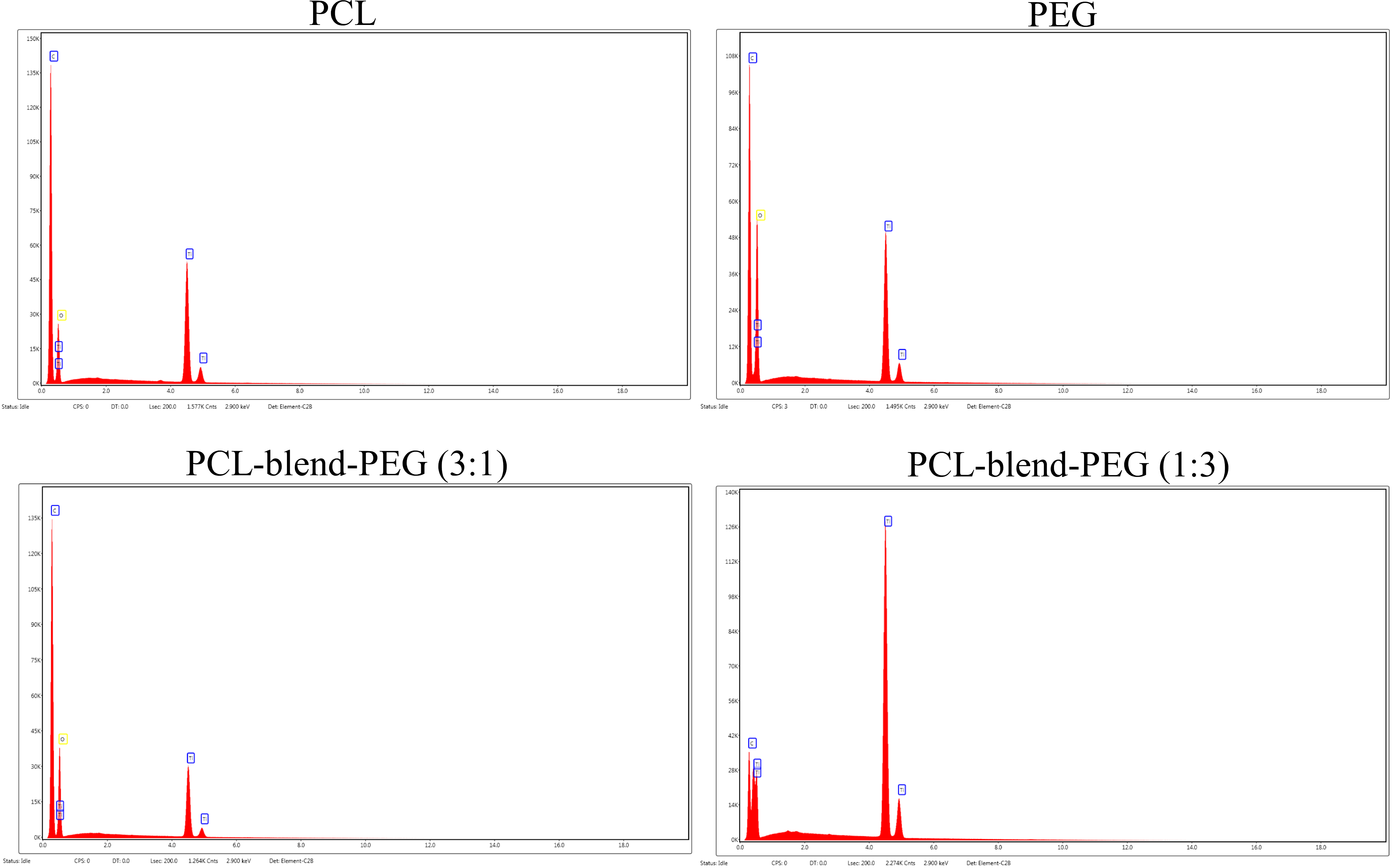

Supplement: Supplementary file 1 [file polymers-12-00717-s001.zip › Supplementary figures/EDS_suplementary.tif]
